# Asylum Construction

**Published:** 1897-12-11

**Authors:** 


### The Institutional Workshop.

### ASYLUM CONSTRUCTION.

(*Continued from page* 176.)

The principle of treating the insane in a congeries of small asylums or villas scattered over the grounds, instead of in one or two main buildings, and described by Dr. Sibbald as the segregated type, has found its best development in the Prussian asylum at Halle. It is not quite the same as the village colony at Ghed; but it approximates to it, and the system has been tried to some extent in Scotland, and to a still smaller extent in a few English asylums. Very often hospitals which were built for infectious diseases have been used as homes for a dozen or more harmless cases, and occasionally the buildings at the farm have been made to contain a larger number of working patients; but, so far as we know, there is not any asylum in Great Britain carried out on the lines of the one at Halle. Indeed, we fear that the majority of English superintendents would not approve of the idea, or would look on it with some amount of apprehension. For instance, it might be asked, Who has charge of these villas? When and by whom are the master locks put on in the evening? Is each villa left to itself for ten hours out of the twenty-four? Is there a medical officer resident in each of the villas for the sick and acute? What about visits of the night attendants? Where is the cooking carried out? How is the distribution of food effected? Moreover, we doubt whether English patients would like it. Some twenty years since the writer of this article had a ward for ten patients on each side of the house fitted up as a day-room. It was arranged so that patients might enter it and leave it when they wished. They were allowed perfect freedom, and no attendant was in the room with them. It was found impossible to keep up the number to ten, as the patients greatly preferred the life and bustle of a large ward, and the idea had to be given up.

In spite of the theoretical objections which might be raised against the Halle system it has something seductive about it, and if it could be transplanted to England and successfully worked, then theoretical objections must be given to the winds. London's lunatics increase by about 700 a year. Why should not the County Council try the principle for one year's increment of their insane?

We shall now give the reader an idea of the Prussian asylum, following Dr. Sibbald's admirable description as closely as our space will permit. The asylum contains 960 patients, and it may be said to be divided into two main sections, something like the Gartloch Asylum, but here all resemblance ends. One of the sections is known as the medical section and the other as the colony. The medical section consists of sixteen blocks. Six of these are arranged in the form of a square. In the south front is the steward's department, and on the north are the pathological rooms. Four blocks, shaped like the letter L, occupy the corners of the square. These blocks accommodate the untrustworthy patients (36 of each sex) and the probationary patients (37 of each sex), the probationary patients meaning those likely to be soon transferred to the colony. The hospital is in the centre of the square and contains 18 patients of each sex, and it is the only block containing both men and women. On each side of the square are three blocks arranged in triangles. One of these is called the observation division for private female patients, and another the observation division for female pauper patients, and the third is the closed division for violent female patients who are constantly under lock and key. The three blocks on the opposite side of the square contain the corresponding classes of male patients. South-east of these are three blocks, one for feeble men, another for feeble women, and a third contain the offices of the officials. The high road from Halle to Leipsic passes close to these blocks. On the opposite side of the road are the kitchen buildings, the laundry, five villas for women, the dairy, with accommodation for women, the assembly rooms, the medical superintendent's house, seven villas for men, workshops, farm, and industrial buildings. All these, numbering about 35 distinct blocks, contain between 25 and 50 patients each. There are no walls surrounding any part of the estate, but palings surround those blocks wherein the worst patients are warded.

The architecture is quite plain, and an old village has been incorporated in the colony "and tends greatly to make one forget it is an asylum for the insane."

